# The effect of demand-side financial incentives for increasing linkage into HIV treatment and voluntary medical male circumcision: A systematic review and meta-analysis of randomised controlled trials in low- and middle-income countries

**DOI:** 10.1371/journal.pone.0207263

**Published:** 2018-11-14

**Authors:** Augustine T. Choko, Sophie Candfield, Hendramoothy Maheswaran, Aurelia Lepine, Elizabeth Lucy Corbett, Katherine Fielding

**Affiliations:** 1 TB/HIV Theme, Malawi-Liverpool-Wellcome Clinical Research Programme (MLW), Blantyre, Malawi; 2 London School of Hygiene & Tropical Medicine (LSHTM), London, United Kingdom; 3 Department of Public Health and Policy, University of Liverpool, Liverpool, United Kingdom; Johns Hopkins University, UNITED STATES

## Abstract

**Introduction:**

Linkage to HIV treatment is a vital step in the cascade of HIV services and is critical to slowing down HIV transmission in countries with high HIV prevalence. Equally, linkage to voluntary medical male circumcision (VMMC) has been shown to decrease HIV transmission by 60% and increasing numbers of men receiving VMMC has a substantial impact on HIV incidence. However, only 48% of newly diagnosed HIV positive people link to HIV treatment let alone access HIV prevention methods such as VMMC globally.

**Methods:**

A systematic review investigating the effect of demand-side financial incentives (DSFIs) on linkage into HIV treatment or VMMC for studies conducted in low- and middle-income countries. We searched the title, abstract and keywords in eight bibliographic databases: MEDLINE, EMBASE, Web of Science, Econlit, Cochrane, SCOPUS, IAS Conference database of abstracts, and CROI Conference database of abstracts. Searches were done in December 2016 with no time restriction. We fitted random effects (RE) models and used forest plots to display risk ratios (RR) and 95% CIs separately for the linkage to VMMC outcome. The RE model was also used to assess heterogeneity for the linkage to HIV treatment outcome.

**Results:**

Of the 1205 citations identified from searches, 48 full text articles were reviewed culminating in nine articles in the final analysis. Five trials investigated the effect of DSFIs on linkage to HIV treatment while four trials investigated linkage to VMMC. Financial incentives improved linkage to HIV treatment in three of the five trials that investigated this outcome. Significant improvements were observed among postpartum women RR 1.26 (95% CI: 1.08; 1.48), among people who inject drugs RR 1.42 (95% CI: 1.09; 1.96), and among people testing at the clinic RR 1.10 (95% CI: 1.07; 1.14). One of the two trials that did not find significant improvement in linkage to ART was among people testing HIV positive in clinics RR 0.96 (95% CI: 0.81; 1.16) while the other was among new HIV positive individuals identified through a community testing study RR 0.82 (95% CI: 0.56; 1.22).

We estimate an average 4-fold increase in the uptake of circumcision among HIV negative uncircumcised men from our fitted RE model with overall RR 4.00 (95% CI: 2.17; 7.37). There was negligible heterogeneity in the estimates from the different studies with I-squared = 0.0%; p = 0.923.

**Conclusions:**

Overall, DSFIs appeared to improve linkage for both HIV treatment and VMMC with greater effect for VMMC. Demand-side financial incentives could improve linkage to HIV treatment or VMMC in low- and middle-income countries although uptake by policy makers remains a challenge.

## Introduction

There are approximately 1.1 million deaths every year due to HIV infection worldwide with an estimated 1.9 million annual incident infections, the majority in low- and middle-income countries (LMIC)[1]. Nearly 40% of people living with HIV (PLWH) who were newly diagnosed HIV positive were reported to link into HIV care in a recent systematic review [2]. The benefits of timely initiation of ART [3] and effective HIV prevention including voluntary medical male circumcision (VMMC) [4,5,6,7] have changed the emphasis of HIV testing and services (HTS) from learning one’s status to appropriate linkage and retention [1]. Uptake of HTS and linkage into care or prevention remains below current targets [8] in most LMIC [9]. Modeling studies showed that circumcising 80% of HIV negative men aged 15-49y within 15 years would avert 3.4 million incident infections [10]. However, in priority countries, only up to 30% of this target was reached in 2013 showing an estimated gap of 50% for uptake of VMMC [10].

Recent UNAIDS targets aim to ensure that 90% of all PLWH are aware of their HIV status, with 90% of those found HIV positive started onto ART [8]. Efforts to increase access to HTS also provide an opportunity to ensure those men found HIV negative are offered VMMC. Of more concern has been the finding that whilst HIV incidence decreased before 2010, it has remained static since [11], highlighting the need to increase uptake of effective HIV treatment and prevention strategies. One approach to increasing demand for HIV treatment and prevention is through the use of financial incentives.

Financial incentives (FI) are a potential strategy for increasing demand for health services by compensating users’ direct (e.g. transport) and indirect costs (e.g. opportunity cost of time) [12]. Conditional FIs require a pre-specified action before receipt of the incentive [12], and thus psychologically nudge individuals to prioritize health service utilization [13]. Recently there has been significant interest in LMICs on using demand-side financial incentives (DSFIs) to encourage desirable public health behaviours including for HIV [14]. DSFIs are incentives offered to a specified target population (as opposed to providers of goods or services) with the aim of increasing demand for goods or services of merit to that population [15]. Evidence in the literature suggests DSFIs can lead to increased use of preventive and treatment services [16]. The use of DSFIs may be contributory to meet HIV treatment and prevention goals in LMICs. We therefore conducted a systematic review and meta-analysis to investigate the effect of DSFIs as an intervention in LMICs for increasing linkage into HIV treatment or VMMC. We have not identified a meta-analysis specifically summarizing the effect of incentives on linkage to care or VMMC thus far.

## Materials and methods

A systematic literature review and meta-analysis of published and unpublished trials (PROSPERO 2015: CRD42015029248) was conducted [17]. For a trial to be eligible it had to be an individually randomised controlled trial (RCT) or a cluster randomised trial (CRT) undertaken in a low- and middle-income country (LMIC). There was no time or language restriction. The review was restricted to LMICs because the epidemiology and management of HIV, as well as the economic conditions in the region, differ substantially to other settings. Trials were separated into two broad categories namely; studies investigating linkage into HIV treatment (ART) and linkage into VMMC as two primary outcomes for this systematic review, and a trial was included if it reported either of these two outcomes. These outcomes were not necessarily primary outcomes in the original studies. We defined linkage to care as newly diagnosed HIV positive patients making at least one clinic visit where they could have been assessed for ART eligibility and start ART within a specified period of time. Linkage to VMMC was defined as undergoing the procedure within a specified period of time as indicated by the study investigators. The rationale for choosing these two outcomes was that ART and VMMC are the only proven effective interventions for reducing HIV transmission for HIV positive and uncircumcised HIV negative men. While there are extant systematic reviews reporting the effect of incentives for retention on ART, we did not find a systematic review investigating the effect of financial incentives on linkage to ART or VMMC.

### Types of interventions

This review included both conditional and unconditional DSFIs. Incentives included cash, goods, vouchers or microfinance such as a loan (Table 1). Trials that offered supply-side financial incentives to health workers, for example to improve their performance, were excluded.

**Table 1:** Some financial incentives and their definitions.

Form of financial incentive	Definition
**Cash**	Hard cash given to the participant directly or indirectly e.g. via mobile money
**Voucher**	Coupon given to participant or their representative to redeem e.g. at a shop, or to buy airtime, or to cover part of the cost of a health good or service
**Goods**	Anything tangible given to the participant e.g. soap, sweets, school uniform
**Microfinance**	Any financial assistance given directly or indirectly to the participants or their representative e.g. loan and school fees

### Information sources and search strategy

Initial searches were done in MEDLINE and Cochrane library to determine if the review question had already been addressed. We searched the title, abstract and keywords in eight bibliographic databases in December 2016, namely: MEDLINE, EMBASE, Web of Science, Econlit, Cochrane library, SCOPUS, international AIDS Society (IAS) and conference on retroviruses and opportunistic infections (CROI) conference databases of abstracts. We also searched the reference lists of all selected papers to see if any studies were missed. Review of abstracts from two main HIV conferences and slides was done for conferences held between 2004 and 2016 to identify unpublished trials. This time restriction was for ease of searching purposes particularly for online published abstract books for conferences. Record identification data were then extracted and entered into an Excel database for initial screening.

**Fig 1:**
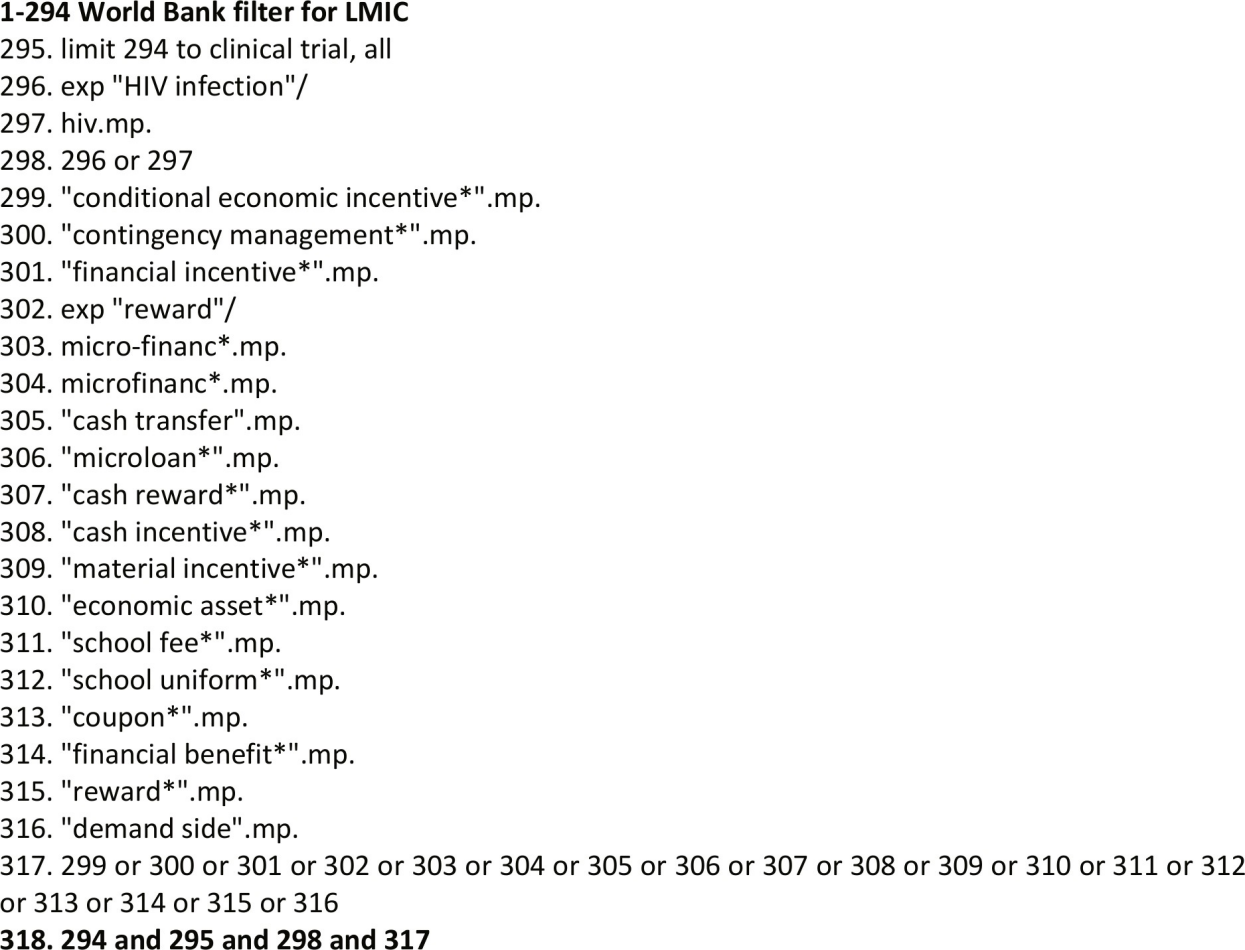
Search Strategy consisting 3 main categories (low and middle income countries; HIV; and financial incentives).

Our search keywords comprised three main categories linked by ‘AND’, with keywords within each category linked by ‘OR’ (Fig 1): keywords to restrict to LMIC using a search filter based on World Bank 2014 definition of LMIC; keywords related to ART and VMMC; keywords related to FIs (Table 1). Appropriate combination of key words and characters were used for each database searched. The search strategy was initially piloted in Medline by AC to determine the best approach.

### Study selection

AC was responsible for running all the searches and removing duplicates from records. Two reviewers (AC and SC) first reviewed titles and abstracts, and removed records that were not relevant before doing full text review. If the abstract was judged by either reviewer to be potentially relevant, a full text review was conducted. For conference abstracts, the presentation slides were accessed from the conference website if available. For inclusion in the final review, both reviewers reviewed all full articles/conference presentations, if both agreed, data were independently extracted and compared by both AC and SC and any discrepancies resolved. A third reviewer (KF) resolved any lack of consensus regarding inclusion of an article.

### Data analysis and quality assessment

**Fig 2:**
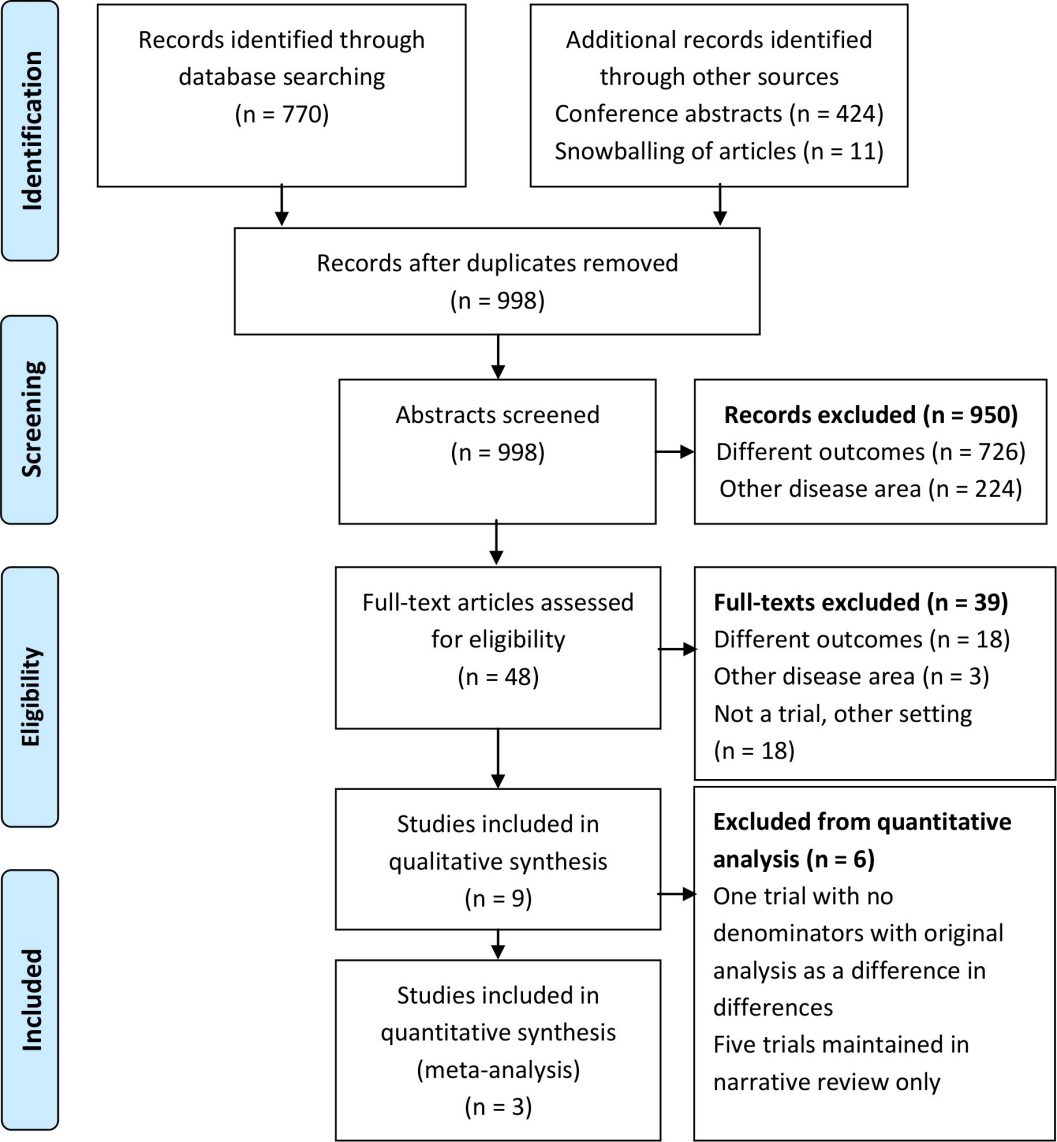
Flow diagram of search results.

A PRSIMA flow diagram was used to provide detailed description of the review process including the number of titles returned to trials that were finally included (Fig 2). Trials are described with respect to setting, study population, outcomes and risk ratios (RRs) and 95% confidence intervals (CI), as presented by the original authors of the trial. For trials that reported odds ratios (OR) we converted these effect estimates into RRs in order to standardize reporting across trials [18]. Meta-analysis was used to combine results from studies to investigate heterogeneity and to obtain a summary RR, comparing interventions with FIs versus no FIs (control). We used a random effects (RE) regression to estimate the summary RR and 95% confidence interval (CI) and used forest plots to display estimates and 95% CIs, separately for each of the two primary outcomes. Heterogeneity across the studies was assessed using the I^2^ statistic from the RE model. We added 0.5 in the numerator for trials with zero outcomes [19]. For trials with multiple intervention arms, we combined into one arm before pooling the estimates using meta analysis [19].

The quality of studies included was assessed by AC and SC using the Downs and Black checklist for randomised studies [20]. This is a score-based assessment tool with five different domains: reporting, external validity, bias, confounding, and power with a maximum score of 32 across the domains.

## Results

### Search results

Of 1205 citations identified from searches in databases, conference abstracts and snowballing titles and abstracts, 998 (82.8%) remained after removal of duplicates (Fig 2). A total of 950 citations were excluded after title and abstract screening because they reported other outcomes (726) or they were in another disease area than HIV (224). A total of 48 articles had a full text review of which nine were finally included in this systematic review (Fig 2). The majority (18/39) of the full text articles excluded were non-randomised studies. Of the nine studies included one was based on a conference abstract.

### Description of included studies

Five trials investigated the effect of DSFIs on linkage to HIV treatment and four trials investigated linkage to VMMC (Table 2). All studies included adult participants (age range: 18–70 years). Six of the nine studies were individually randomised, and all but one trial was undertaken in sub-Saharan Africa. Trial sizes ranged from 86 to 2201 participants. Within a trial the number of comparisons to the control arm ranged from one to five. The majority of trial arms evaluated fixed (n = 10) incentives while some evaluated lottery based incentives (n = 2). Only one trial provided cash incentives [21] while the rest implemented non-cash incentives including mobile airtime as described in the protocol [22,23], food vouchers [24,25], smart phones through raffle draw [26], and subsidy for the VMMC procedure fee [27].

**Table 2:** Characteristics of included studies.

Author, year	Country	Type	Sample size	Arms	Study population		Outcomes	Intervention (s)
**Review outcome: linkage into HIV treatment**								
Maughan-Brown, 2018	South Africa	RCT	86	2	Adults referred for ART Mean age: 33		**PO**: *Linkage to care in 3m* Uptake of treatment following ART referral by a mobile health clinic	US$23 conditional on starting ART <3m
Elul, 2017	Mozambique	CRT	2004	3†		Newly diagnosed HIV positive Median age: 34	**PO:** *Linkage to care in 1m* Retention at 12 months after diagnosis	Combination of: point of care CD4; accelerated ART initiation; SMS reminder; health information; and airtime incentive
Yotebieng, 2016	DR Congo	RCT	433	2		Newly diagnosed pregnant women Age 29 (IQR 25–34)	**PO**: Retention at 6 weeks postpartum Uptake of PMTCT services *Acceptance of proposed services postpartum‡*	US$5, plus US$1 increment at every subsequent clinic visit
McNairy, 2016	Swaziland	CRT	2201	2		Newly diagnosed HIV positive Age 32 (IQR 26–40)	*Linkage to care in 1 month* **PO:** Retention in care at 12m	Combination of: point of care CD4; accelerated ART initiation; phone reminder; health information; and airtime incentive
Solomon, 2014	India	RCT	120	2		HIV positive Injecting drug users Age 38 (IQR 32.5–44)	**PO:** *Linkage to care in 1m* Clinic visit in 12m eg refill HIV RNA suppression at 12m	Control: Voucher incentives through lottery Intervention: Target-based voucher incentives ($4 for linkage, max $48 over 12m)
**Review outcome: linkage into voluntary medical male circumcision (VMMC)**								
Thirumurthy, 2016	Kenya	RCT	909	3		Uncircumcised men Age 29.0 (SD: 6.0)	**PO:** *VMMC uptake in 3m*	Intervention 1: Food voucher ($12.5) Intervention 2: Lottery equivalent of $12.50
Bazant, 2016 *	Tanzania	CRT	*	2		Uncircumcised men Age 27.6 (SD: 9.7)	**PO:** *Number of VMMCs in 3m*	Lottery: weekly smartphone raffle
Thirumurthy, 2014	Kenya	RCT	1502	4		Uncircumcised men Age range: 25–49	**PO:** *VMMC uptake in 2m*	Fixed incentives: $2.5, $8.75, $15.0
Chinkhumba, 2014	Malawi	RCT	1634	6		Uncircumcised men Age 26.7 (SD: 5.8)	**PO:** *VMMC uptake in 6m*	Fixed subsidy for VMMC procedure: (Full subsidy $6, $5.67, $5.3, $4.67, $2.67, $0)

RCT: randomized controlled trial; CRT: cluster randomized trial; PMTCT: prevention of mother to child transmission; PO: primary outcome used by the original authors (Italic: outcome used in this review); SD: standard deviation.

† Trial arm without a financial incentive not used in this review.

‡ Includes CD4 count testing.

* Trial not included in any further analysis due to lack of denominator data. Original analysis as difference in differences.

In two studies, investigators provided a larger fixed amount incentive (e.g US$ 5) initially, followed by a smaller (e.g. US$ 1) incremental incentive conditional on clinic attendance or attainment of pre-specified goals [21,28]. In two trials [23,29], investigators compared the control arm to a complex intervention consisting of five components: point of care CD4; accelerated ART initiation; telephone reminder; health information; and non-cash incentives (mobile airtime) [22,23]. One trial, conducted among people who inject drugs (PWID), compared two incentives: the control arm offered a lottery prize equivalent to $4 with eligibility for the lottery not conditional on meeting treatment targets, whilst the intervention arm offered non-cash vouchers (US$4–8) conditional on meeting HIV treatment targets [28].

### Quality assessment

Quality of the trials included were assessed as generally good, with Downs and Black scores ranging from 19–29 out of a maximum of 32 (Table 3). For the unpublished study only the abstract and conference presentation slides were assessed and we were unable to assess for blinding, adverse events, loss to follow-up and adjustment for potential confounding factors which resulted in a lower score [29]. Solomon *et al*. 2014 [28] had a slightly lower score (28) due to potential selection bias because participants were drawn from individuals (drug users) available to outreach providers. Such sampling frame may not represent the source population as the majority of HIV patients who inject drugs in LMICs may not be in this patient category. Chinkhumba *et al*. 2014 [27] had the lowest quality score of 16 because only p-values were reported with no numerators and denominators and intervention effect estimates. Furthermore, there was no sample size section reported.

**Table 3:** Quality assessment of the included studies.

	Assessment domains					
Author, year	Reporting	External validity	Bias	Confounding	Power	Maximum Score
Yotebieng 2016	9	3	6	6	5	29
McNairy, 2016	8	3	5	5	5	26
Solomon 2014	9	3	6	6	4	28
Thirumurthy, 2016	9	3	5	6	5	28
Bazant, 2016*	8	3	4	4	4	23
Thirumurthy, 2014	10	3	5	6	5	29
Chinkhumba, 2014	5	3	7	4	0	19

**f** Possible total score: Reporting (11); external validity (3); bias (7); confounding (6); power (5). Rated using the Downs and Black checklist.

* Trial not included in meta-analysis (Table 4) due to lack of denominator data. Original analysis as difference in differences

### Linkage into HIV treatment

Of the 5 linkage to ART trials, 2 involved newly diagnosed HIV positive adults at the clinic; one was among pregnant women attending postpartum services, one was among individuals testing HIV positive during mobile HIV testing [30], and the final one was among PWID (Table 2). Three trials found that FIs significantly improved linkage to ART as reported by the original authors (Table 4). A FI significantly improved linkage to ART among women postpartum in DR Congo (RR 1.26, 95% CI: 1.08; 1.48), as it did among PWID testing positive in an outreach activity in India (RR 1.42, 95% CI: 1.09; 1.96). In Swaziland, a combination strategy including a FI significantly improved linkage to HIV treatment by 10% (RR 1.10, 95% CI: 1.07; 1.14) among patients diagnosed at the clinic.

**Table 4:** Trial outcomes: Linkage to ART and voluntary male medical circumcision.

Author, year	Country	Type	Sample size	Arms	Study population	Outcomes	Intervention (s)
**Review outcome: linkage into HIV treatment**							
Maughan-Brown, 2018	South Africa	RCT	86	2	Adults referred for ART Mean age: 33	**PO**: *Linkage to care in 3m* Uptake of treatment following ART referral by a mobile health clinic	US$23 conditional on starting ART <3m
Elul, 2017	Mozambique	CRT	2004	3†	Newly diagnosed HIV positive Median age: 34	**PO:** *Linkage to care in 1m* Retention at 12 months after diagnosis	Combination of: point of care CD4; accelerated ART initiation; SMS reminder; health information; and airtime incentive
Yotebieng, 2016	DR Congo	RCT	433	2	Newly diagnosed pregnant women Age 29 (IQR 25–34)	**PO**: Retention at 6 weeks postpartum Uptake of PMTCT services *Acceptance of proposed services postpartum‡*	US$5, plus US$1 increment at every subsequent clinic visit
McNairy, 2016	Swaziland	CRT	2201	2	Newly diagnosed HIV positive Age 32 (IQR 26–40)	*Linkage to care in 1 month* **PO:** Retention in care at 12m	Combination of: point of care CD4; accelerated ART initiation; phone reminder; health information; and airtime incentive
Solomon, 2014	India	RCT	120	2	HIV positive Injecting drug users Age 38 (IQR 32.5–44)	**PO:** *Linkage to care in 1m* Clinic visit in 12m eg refill HIV RNA suppression at 12m	Control: Voucher incentives through lottery Intervention: Target-based voucher incentives ($4 for linkage, max $48 over 12m)
**Review outcome: linkage into voluntary medical male circumcision (VMMC)**							
Thirumurthy, 2016	Kenya	RCT	909	3	Uncircumcised men Age 29.0 (SD: 6.0)	**PO:** *VMMC uptake in 3m*	Intervention 1: Food voucher ($12.5) Intervention 2: Lottery equivalent of $12.50
Bazant, 2016 *	Tanzania	CRT	*	2	Uncircumcised men Age 27.6 (SD: 9.7)	**PO:** *Number of VMMCs in 3m*	Lottery: weekly smartphone raffle
Thirumurthy, 2014	Kenya	RCT	1502	4	Uncircumcised men Age range: 25–49	**PO:** *VMMC uptake in 2m*	Fixed incentives: $2.5, $8.75, $15.0
Chinkhumba, 2014	Malawi	RCT	1634	7	Uncircumcised men Age 26.7 (SD: 5.8)	**PO:** *VMMC uptake in 6m*	Fixed subsidy for VMMC procedure: (Full subsidy $6, $5.67, $5.3, $4.67, $2.67, $0)

RCT: randomized controlled trial; CRT: cluster randomized trial; PMTCT: prevention of mother to child transmission; PO: primary outcome used by the original authors (Italic: outcome used in this review); SD: standard deviation, m: month.

† Trial arm without a financial incentive not used in this review.

‡ Includes CD4 count testing.

* Trial not included in any further analysis due to lack of denominator data. Original analysis as difference in differences.

**Fig 3:**
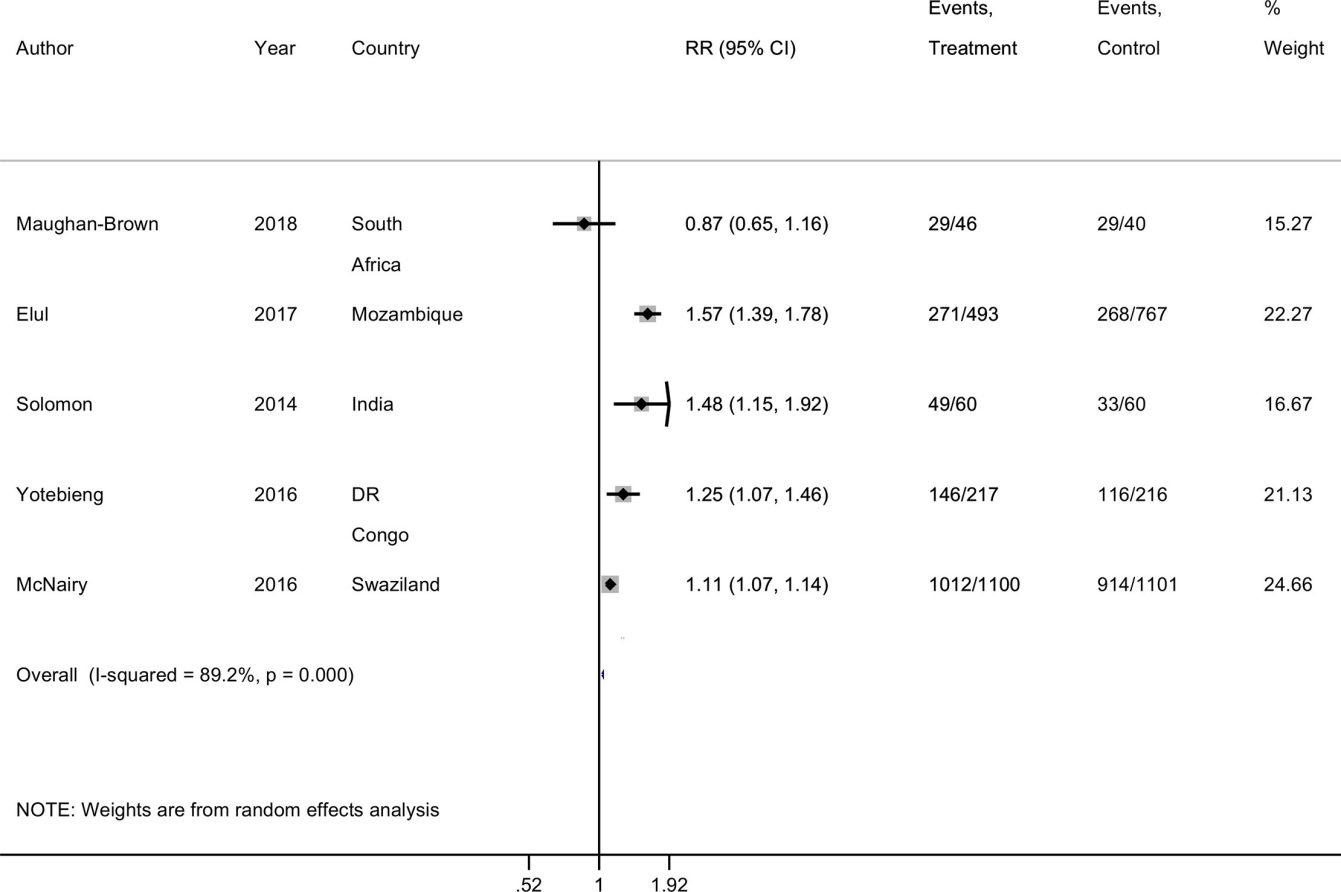
Forest plot of linkage to HIV treatment estimates. From meta-analysis of the data reported by the original authors (Table 4). Form: mode of giving financial incentive.

Two trials found that FIs did not significantly improve linkage to ART. One was among people testing HIV positive in clinics in Mozambique (RR 0.96, 95% CI: 0.81; 1.16) while the other was among newly diagnosed HIV positive individuals identified through a community testing study (RR 0.82, 95% CI: 0.56; 1.22). There was considerable heterogeneity between the five trials reporting on linkage to ART (I-squared of 89.2%, p < 0.001) and differences in study populations, and so a pooled estimate is not reported (Fig 3).

### Linkage into voluntary male medical circumcision (VMMC)

Among HIV negative men, fixed FIs appeared to improve the proportion of men undergoing circumcision as reported by the original authors (Table 4). The proportion of HIV negative men who had circumcision was generally low in the studies with uptake ranging from 0.7% to 8.4% in intervention arms and 0–1.6% in control arms, over a follow-up period ranging from 2 to 6 months. However, the effect appeared stronger for higher compared with smaller values of incentives [24]. For example, while USD15.0 improved uptake of circumcision within 2 months in Kenya, USD2.5 did not (RR 5.72,95% CI: 2.54; 12.25 and RR 1.10, 95% CI: 0.40; 3.18, respectively) [24]. One study had a lottery FI arm (mean value of $12.5), as well as a fixed FI intervention arm, but in contrast to the fixed FI, the lottery FI did not significantly improve the uptake of VMMC within 3 months aRR 2.45 (95% CI: 0.80; 7.42) [25]. A trial offering five varying subsidies for the circumcision procedure to uncircumcised men found that offering full subsidy (free circumcision) increased uptake from 0.0% without subsidy to 3.0% [27] (Table 4).

One study was not included in meta-analysis because there were no denominators and the outcome was VMMC increases analysed as a difference in differences that did not fit with the overall analytical approach [26].

**Fig 4:**
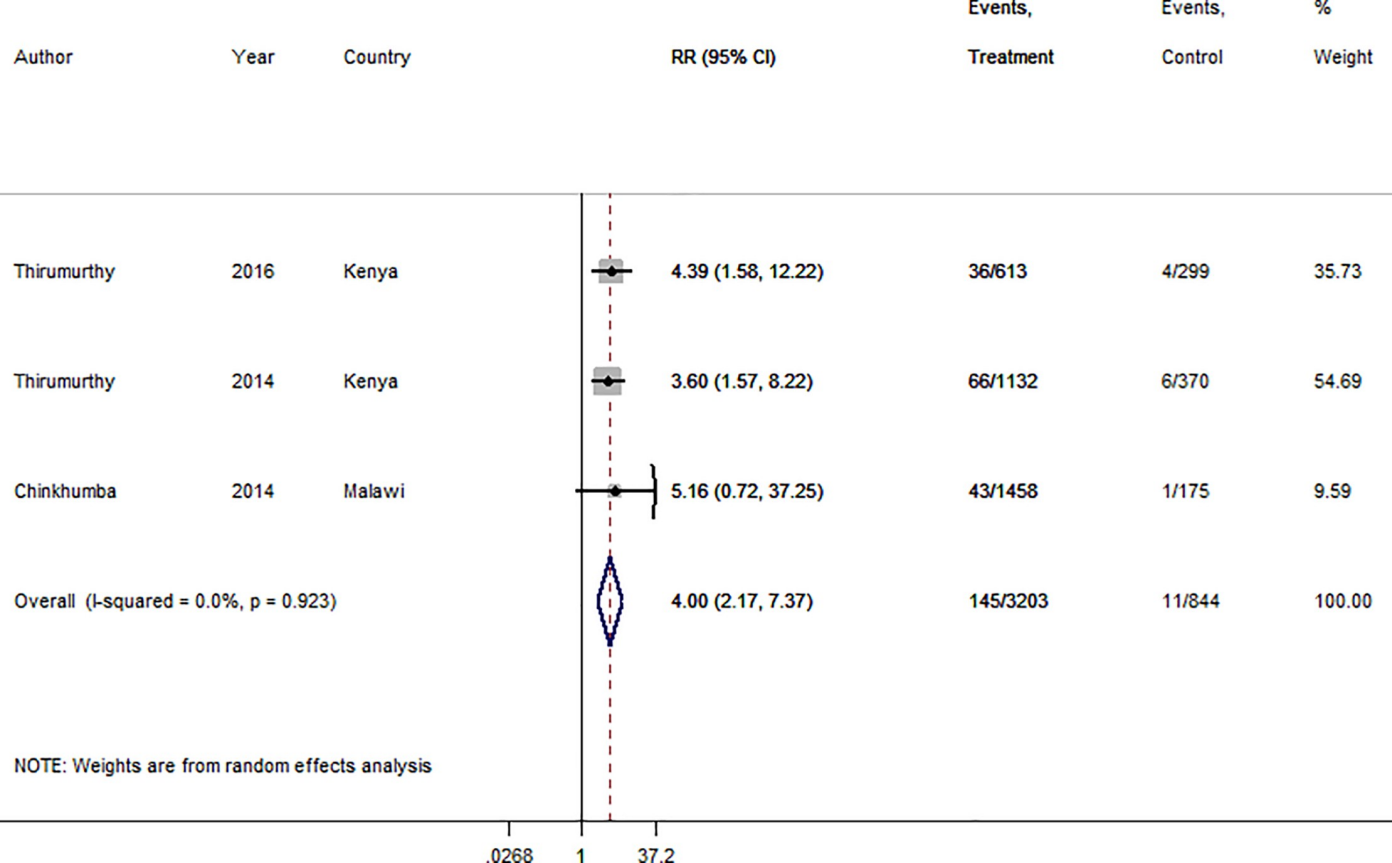
Forest plot of linkage to voluntary medical male circumcision (VMMC) estimates. From meta-analysis of the data reported by the original authors (Table 4). Form: mode of giving financial incentive.

We estimate an overall 4-fold increase in the uptake of circumcision among HIV negative uncircumcised men from our fitted RE model (Fig 4) with overall RR 4.00 (95% CI: 2.17; 7.37). There was negligible heterogeneity in the estimates from the different studies with I-squared = 0.0%; p = 0.923.

## Discussion

The main finding from this systematic review was that demand-side financial incentives (DSFIs) seemed to improve linkage to voluntary medical male circumcision (VMMC) but with unclear effect on linkage to HIV treatment in low and middle income countries. It is unclear why financial incentives worked better for the VMMC outcome compared to the linkage to ART outcome. The age and sex profile of participants in the included trials was very similar (Table 5), although all three VMMC trials among men reported significant improvement. A potential explanation is that the targeted study populations may perceive VMMC differently from linkage to ART. While linkage to ART implies a lot of additional steps some of which may imply disclosure of being HIV positive, VMMC is a one-off procedure. This implies that VMMC uptake may be higher as it is not associated with stigma in the same way linkage to ART may be. We note that although FIs increased linkage to VMMC, the numbers were still very small, for example absolute differences were small despite large relative risks.

**Table 5:** Characteristics of participants in the included studies.

	Male	Female	Age	Employed?	
Author, year	n (%)	n (%)	Estimate (variation)	Yes (n, %)	Type of incentive
**Linkage to HIV treatment trials**					
Maughan-Brown, 2018	31 (36.0)	55 (64.0)	Mean: 33.0	28 (32.6%)	Voucher
Elul, 2017	712 (36.0)	1,292 (64.0)	Median: 34.0	1,473 (74.0)	Air time
Yotebieng, 2016	0 (0.0)	433 (100)	29 (IQR 25–34)	Not reported	Cash
McNairy, 2016	903 (41.1)	1,294 (58.9)	31 (IQR 26–39)	1042 (47.4)	Air time
Solomon, 2014	109 (90.8)	11 (9.2)	Median: 38	102 (85.0)	Voucher
**Linkage to HIV voluntary medical male circumcision trials**					
Thirumurthy, 2016	909 (100)	0 (0.0)	Mean: 29 (SD: 5.9)	Not reported	Food voucher
Thirumurthy, 2014	1502 (100)	0 (0.0)	Mean: 34.4 (SD: 6.7)	Not reported	Food voucher
Chinkhumba, 2014	1634 (100)	0 (0.0)	Mean: 26.7 (SD: 5.8)	Not reported	Subsidy

Most studies provided fixed FIs (cash, food vouchers, mobile airtime) of varying levels and only one study had a lottery arm. We identified few studies that investigated the use of FIs to improve linkage to HIV treatment or VMMC in LMICs, and the amount of the incentive was found to determine effectiveness. The findings highlight the value of using FIs for increasing demand for HIV treatment and prevention services in the region, especially where healthcare providers may be exploring approaches to rapidly scale up coverage of these services.

The WHO recently emphasized the need for closer integration of HIV prevention services to HIV testing services, particularly VMMC and pre-exposure prophylaxis (PrEP) [1]. VMMC has been shown to be potentially cost saving at scale [31] and ensuring high levels of coverage in countries facing a generalized HIV epidemic has the potential to avert millions of new HIV infections [31]. However, recent estimates suggest over 20 million young adults are still to be reached by VMMC services in high priority countries [11]. The evidence from this systematic review suggests there is a potential role of DSFIs to meet these goals in LMICs hardest hit by the HIV pandemic [1]. VMMC does not require adults to attend health facilities multiple times over long-time periods, which may explain why DSFIs were found to increase demand.

Previous studies have found FIs may be more effective for simpler than complex behavioral change [32,33]. The value of incentives given in the studies reviewed ($2.50 to $15.0) was considerably lower than previously estimated costs for performing VMMC ($ 75–95 in 2010 prices) [31]. Although providing FIs may increase VMMC intervention costs, the resulting increase in uptake of VMMC may still result in the intervention being cost-effective. It is important to note that even with FIs, VMMC uptake only increased by 9%, implying that other strategies than FIs may be needed to address residual barriers [27]. Thirumurthy *et*. *al* [24] and Chinkhumba *et*. *al* [27] provide important insight into the dosing effect of FIs. The authors found that unless the incentive exceeded a certain threshold value, it was not found to be effective. This suggests that careful assessment of the size of the incentive on offer is warranted if incentives are to be effective.

We were intrigued by the apparent lack of effect by lottery-type of incentives as these have been shown to work in other settings albeit in different disease areas [34]. Lottery-based FIs have also been shown to reduce HIV incidence within the African region by influencing sexual behaviour patterns [35]. One study–and only one arm investigated this intervention, hence more studies may be required to understand the impact of lottery interventions. The theory behind lottery is that individuals tend to overestimate the probability to win and thus are more likely to take the risk [36].

Although only three of the five trials investigating DSFIs showed improvement in linkage to care, we note that the actual linkage proportions were high in the intervention arms of the trials: 55.0–92.0% [21,28,29], and in control arms with range: 34.9–83.0%. It was interesting to note that among PWID, a fixed incentive was more effective than the control with a lottery intervention at increasing linkage. Furthermore, 50% of participants receiving the fixed incentive linked to clinic within 5 days of randomisation [28]. This is an important finding as it suggests that DSFIs can be used to improve rapid linkage [37] which may lead to earlier viral suppression and therefore long term good health, especially in patients with low CD4 count (<500 cells/μ) [38].

There are a number of limitations with this systematic review. First, we broadly categorized incentives as fixed or lottery which may lack directness on what specific type of incentive (cash, voucher, gift, or subsidy) may be more effective. However, a recent comparison of cash vs voucher incentives concluded that cash incentives were more preferred by both the program and recipients in a humanitarian aid context in DRC [39]. Second, for linkage to VMMC, outcomes were measured over different follow-up periods, an element not accounted for in the analysis. Third, we included one trial whose linkage to HIV treatment outcome only included attendance for CD4 count which may not necessarily mean initiation of HIV treatment. Fourth, we included two trials offering a combination of several interventions including a financial incentive. As the financial incentive was only a part of the combination strategy, it may not be possible to isolate the incremental effect of the financial incentive alone. Finally, some trials may have been missed because the search key words did not include ART, or because the primary outcome of the review was not the primary outcome of the original trials.

HIV testing is seen as key to ensuring universal access to HIV treatment and prevention services, however, linkage into these services after HIV testing remains sub-optimal. In this systematic review we found the use of DSFIs significantly improved linkage into HIV treatment and the uptake of VMMC in LMICs. As HIV testing services are being scaled-up across LMICs to meet UNAIDS targets, healthcare providers in countries where ART and VMMC coverage remains low may need to consider offering FIs to increase demand. Further work is needed to explore the use of FIs along the HIV cascade of services from testing, linkage to viral suppression.

## Supporting information

S1 Checklist PRISMA_Checklist. (DOC)
